# Expansion of the agricultural frontier in the largest South American Dry Forest: Identifying priority conservation areas for snakes before everything is lost

**DOI:** 10.1371/journal.pone.0221901

**Published:** 2019-09-10

**Authors:** María Soledad Andrade-Díaz, Juan Andrés Sarquis, Bette A. Loiselle, Alejandro R. Giraudo, Juan Manuel Díaz-Gómez

**Affiliations:** 1 Instituto de Bio y Geociencias del Noroeste Argentino (Consejo Nacional de Investigaciones Científicas y Técnicas—Universidad Nacional de Salta), Rosario de Lerma, Salta, Argentina; 2 Instituto Nacional de Limnología (Consejo Nacional de Investigaciones Científicas y Técnicas–Universidad Nacional del Litoral), Ciudad Universitaria, Santa Fe, Argentina; 3 Department of Wildlife Ecology and Conservation, Center for Latin American Studies, University of Florida, Gainesville, FL, United States of America; 4 Facultad de Humanidades y Ciencias (Universidad Nacional del Litoral), Ciudad Universitaria, Santa Fe, Argentina; Texas State University, UNITED STATES

## Abstract

Conservation planning relies on integrating existing knowledge, social-environmental contexts, and potential threats to identify gaps and opportunities for action. Here we present a case study on how priority areas for conservation can be determined using existing information on biodiversity occurrence and threats. Specifically, our goals are: (1) to model the ecological niche of twelve endemic snake species in the Dry Chaco Forest, (2) to quantify the impact of the deforestation rates on their distributions, (3) to propose high priority areas for conservation in order to improve the actual protected area system, and (4) to evaluate the influence of the human footprint on the optimization of selected priority areas. Our results demonstrate that Argentinian Dry Chaco represent, on average, ~74% of the distribution of endemic snake species and deforestation has reduced suitable areas of all snake species in the region. Further, the current protected areas are likely insufficient to conserve these species as only very low percentages (3.27%) of snakes’ ranges occur within existing protected areas. Our models identified high priority areas in the north of the Chaco forest where continuous, well-conserved forest still exists. These high priority areas include transition zones within the foothill forest and areas that could connect patches of forest between the western and eastern Chaco forest. Our findings identify spatial priorities that minimize conflicts with human activities, a key issue for this biodiversity hotspot area. We argue that consultation with stakeholders and decision-makers are urgently needed in order to take concrete actions to protect the habitat, or we risk losing the best conservation opportunities to protect endemic snakes that inhabit the Argentinian Dry Chaco.

## Introduction

The loss and fragmentation of native forests due to the expansion of the agricultural frontier have affected the abundance, diversity and distribution of species in tropical and subtropical ecosystems [1–3]. At a global scale, the expansion and intensification of agriculture [4,5] were achieved by prioritizing economic and political interests. In contrast, ecological attributes, such as distinctive habitats, species richness and population abundances, connections between the different patches of remaining forest, among others, often were not considered when decisions were made regarding transformation of land. The failure to consider the maintenance of structure and functionality of forests' ecosystems in environmental policies and decision-making regarding development may result in the loss of socio-economic benefits that these natural ecosystems provide to human populations [6,7]. Moreover, the existing protected areas in Latin America are insufficient and generally, they were not designed focusing on biodiversity conservation goals [8]. Currently, in many places the expansion of the agricultural frontier has enclosed protected areas, thereby transforming them into ‘islands’ where plant and animal populations become isolated [9]. It is in this dynamic scenario of changing land use and inadequate protected area systems, where studies are needed to predict species distributions in order to identify priority areas for conservation and propose effective strategies for their long-term protection.

The tropical dry biomes, currently considered among the most worldwide endangered ecosystems, exemplifies a system where land use changes are rampant and existing protected areas are insufficient to achieve conservation objectives. Further, these biomes have received relatively little attention from both ecologists and conservationists [10–12]. In fact, the few studies focused on the identification of conservation areas for these ecosystems showed that current representativeness levels (i.e., represent the range of expected biological variation) are inadequate, including the tropical seasonally dry forests [13], the Cerrado [14,15] and the Gran Chaco [16]. The ‘Gran Chaco’ is the second largest forest in South America, after the Amazon rainforest [17]. This ecoregion corresponds to Chaco Biogeographic Province encompassing both the Dry and Humid Chaco sub eco-regions [18,19] and it extends through Argentina, Bolivia, Paraguay and southwestern Brazil [20]. From an ecosystem service perspective, this forest is extremely important as it harbors one of the largest extra-tropical carbon stocks in the southern hemisphere [21–23]. Despite being characterized by heterogeneous environments and high species diversity [20], this forest is currently considered as one of the world’s most threatened wooded subtropical ecosystems as a consequence of an intense anthropogenic disturbance as a consequence of logging and agricultural activities [12,22,24,25]. In fact, the main driver of high deforestation rates (200,000 ha/year -1) in the Chaco forest is agribusiness expansion [12,26]. This transformation produces high carbon emissions [27], landscape configuration changes [28], habitat degradation [29], and species extinctions [1]. Likewise, despite the fact that Argentina has the largest area of Chaco (approx. 62% of the total area) [26], we observed that only 1.7% of the Dry Chaco is within the current Protected Areas system (PAs) [30,31]. Moreover, increasing evidence indicates that distribution and survival of inhabiting species on dry ecosystems could also be also affected by climate change which could lead to widespread reduction of current species richness and ecological integrity (e.g. [32–34]). Therefore, in order to identify priority areas and to develop management decisions and conservation strategies, information on species distributions and their vulnerability to land use change is needed [7,35–37].

Establishing a priority areas network that adequately represents the biodiversity within the Dry Chaco with clear conservation goals, while at the same time compatible with the sustained human development is urgently needed [16]. In this sense, different conservation planning schemes have been developed over the last decade [38–40] promoting a representative and connected network of PAs that contributes to the viability of biodiversity and ecosystems [41]. These approaches are based on the distribution of key biodiversity features and anthropic variables, and identifying the most important sites for conservation and sites, which are compatible with sustainable use [42,43]. From this perspective, the integration of species-level surrogates is necessary to ensure that critical habitats and ecosystems within the region are not missed [13,16,44,45]. Snakes have received little attention in land use changes studies [46,47], despite the fact that habitat loss and degradation are one of the main factors driving declines in reptile populations [48–51]. Some studies have shown that habitat loss and the spatial patterns of remaining vegetation affect the physical condition, as well as the distribution of snakes in the landscape [52,53]. Moreover, snakes are killed by humans, despite their important role as prey and predators in ecosystems [54]. Indeed, unpopular animals like snakes have attracted less attention than charismatic vertebrates [55–57], and as a consequence, conservation action plans rarely consider snakes. While PAs are important to preserve snake communities unaffected by human modifications, few reserves have been established that represent snakes adequately [53,58,59]. The Dry Chaco is not an exception to this scenario. In fact, compared to other taxonomic groups, we know little about the natural history, ecology and distribution of most snake species associated with this region [53]. Thus, analyzing the suitable habitats of endemic snakes in the Dry Chaco forest is a crucial step to evaluate and identify conservation priority areas that require immediate protection actions [47].

Nevertheless, delimiting the distribution of a species is a complex task that involves many determining both factors, both biotic and a biotic, which are difficult to assess through fieldwork [60,61]. Generally, species distribution ranges are frequently represented by polygons [62] and are often used for conservation decisions [16]. However, these approaches suffer from the effects of multi-level conflicts among scales and resolutions and are likely to frequently include many areas not holding populations or exclude others where populations actually occur [60,63]. Thus, data-driven techniques (e.g., Ecological Niche Models [ENMs]) have been developed to predict the potential distribution of species, via identifying suitable areas for species occurrence, as well as pointing to the most relevant bioclimatic variables that predict occurrence [61,64,65].These approaches offer widely accepted methods for summarizing species' distributional patterns for conservation applications [63,66]. Therefore, the use of ENMs and systematic conservation planning methods are useful to achieve conservation goals as they provide critical information on spatial areas required for species conservation [67] and guide conservation decision-making processes regarding actions and policies needed for the long-term protection of biodiversity.

The main objective of this study is to analyze the relationship between the expansion of the agricultural frontier and the distribution of endemic snake species in the Dry Chaco forest. To accomplish this, we use geographical information of 12 snake species endemic to Dry Chaco forest as focal group, and employ ENMs and conservation planning tools. We address the following questions: (1) what are the predicted geographic distributions of the endemic snakes in Argentinean dry Chaco?; (2) which bioclimatic variables that best determine these snake species distributions?; (3) how has agricultural expansion impacted distribution of snake species?; (4) what are the priority areas for conservation of these endemic snakes and what proportion of these areas are represented in the current protected areas system? [68–71]; and, (5) how has human activity (human footprint) influenced the spatial prioritization of an optimal/ representative priority area network?

## Materials and methods

### Occurrence data

We selected 12 snake species, which are endemic to the Dry Chaco forest as focal taxa (Table 1). We obtained occurrence records from reviewing museum collections, reliable literature records and from high-intensity sampling in Argentina for over 28 years (S1 Table). These data were complemented with information obtained from the Global Biodiversity Facility network (GBIF) [72]. Using multiple data sources provided complementarity and served to minimize biases in our data [59,73].Because the information of occurrence records for species has many shortcomings, we filtered the information to avoid inadequate taxonomy and identify problematic or imprecise locality records [74]. We compared the spatial distribution of records obtained with the ranges for species, checking this information with help of specialists (Giraudo pers. obs.), and removed all those mismatched records. For specimens without geographic coordinates, we used Google Earth to define a georeferenced location using information provided with the record. We omitted, however, those records where geographical information could not be verified. We compared these museum and on-line data with expert maps and our knowledge [53] and determined that these data resulted in well-represented geographic coverage of the focal taxa. The combination between expert maps that are in fact an excellent resource for delimiting the broad areas outside which a species is not expected to occur [75] and the well-understood distributions by specialists could be considered the best approach to define empirical geographical distributions [76,77]. Then, using 'ecospat' package in R software [78–80], we removed records repeated in multiple sources and retained only unique localities within a vicinity of 10 km^2^.

**Table 1:** Summary of the bioclimatic variables with their percentage contributions (%) in models predicting snake distribution.

Species	N	Percentage contribution per variable						
		BIO2	BIO4	BIO5	BIO6	BIO13	BIO14	BIO15
***Boa constrictor occidentalis***	44	10	0	70.8	7.6	9.7	0	1.9
***Epicrates alvarezi***	34	23.2	3.3	56.5	12.1	4.8	0	0
***Erythrolamprus albertguentheri***	16	16.5	0	20.9	0	5	9	48.5
***Erythrolamprus sagittifer modestus***	45	0	8.2	77.6	0	2.6	1.1	10.5
***Leptodeira annulata pulchriceps***	15	0	0	100	0	0	0	0
***Philodryas baroni***	63	46.2	3.7	17	16	14.1	0.6	2.4
***Philodryas erlandi***	27	0	0	90	0	8.9	1.1	0
***Phimophis vittatus***	22	5.2	2.1	81.2	0	11.5	0	0
***Psomophis genimaculatus***	6	0	0	84.9	15.1	0	0	0
***Sibynomorphus lavillai***	8	0	0	99.8	0	0.2	0	0
***Thamnodynastes chaquensis***	8	0	0.9	81.4	0.2	17.1	0.4	0
***Xenodon pulcher***	72	0.2	7.3	66.3	3.6	15.9	5.5	1.2

N = number of occurrence records used in models. BIO2 = Mean Diurnal Range (Mean of monthly (max temp—min temp)); BIO4 = Temperature Seasonality (standard deviation *100); BIO5 = Max Temperature of Warmest Month; BIO6 = Min Temperature of Coldest Month; BIO13 = Precipitation of Wettest Month; BIO14 = Precipitation of Driest Month; BIO15 = Precipitation Seasonality (Coefficient of Variation).

### Climatic data

The dry Chaco forest [18] is seasonal with a dry winter and a rainy summer [20]. We obtained data layers from the WorldClim v1.4 at 30” spatial resolution, which includes a set of 19 climatic variables summarizing aspects of precipitation and temperature for the earth's surface for the period 1950–2000 [81]. After a review of the natural history of the species, a jackknife test of variable importance provided by Maxent (i.e. variables with highest gain when used in isolation and variables that decreased the gain the most when they were omitted) and a Pearson correlation analysis between all the variables, we discarded highly correlated variables (r > 0.8) and selected the following seven variables: BIO2 = Mean Diurnal Range; BIO4 = Temperature Seasonality; BIO5 = Maximum Temperature of Warmest Month; BIO6 = Minimum Temperature of Coldest Month; BIO13 = Precipitation of Wettest Month; BIO14 = Precipitation of Driest Month; and BIO15 = Precipitation Seasonality. Layers were prepared and processed using QGIS software v. 2.16.2 [82].

### Ecological niche models

Because dispersal plays a crucial role in the distribution of organisms and should be considered in species modeling [83], we defined an area for model calibration (or M in BAM diagram; [61]) that reflects the accessible historical area for each species. This calibration area was created by a background polygon that corresponded to the extension area for dry Chaco ecoregion. Such consideration assumed that this region represent the species’ tolerance limits, historical barriers to dispersal, and intrinsic need for certain abiotic conditions [84].

We modeled habitat suitability––based on ENM principle––for each species using a maximum entropy method, MaxEnt v3.3.3K [85]. MaxEnt software calculates the potential geographic distribution for each species linking spatial records and bioclimatic variables [85,86]. Although recent studies have shown that there are uncertainties when forecasting species distributions depending on the algorithm used [87,88], we decided to use MaxEnt over other available methods given its proven high performance and suitability for presence-only data [64,89,90]. We used 'ENMeval' package [91] to perform a calibration protocol assessing the model complexity. For this step, models for species with small data sets (<10 records), we applied the n-1 jackknife method proposed by Pearson et al. [89], where each occurrence was used for testing once, while the other records were used to train the model [89,91,92]. For species with more than 10 records, we used an equivalent of the 'cross-validate' method in MaxEnt software, where the 'random k-fold' method partitions occurrence localities randomly into a specific number of bins (in this case we used k = 5 bins). We ran models (using randomly sub-sampled 50% of the data as testing) under varying model response types (feature classes: L, Q, LQ), different values of regularization multiplier values (RM: 1, 2.2, 4.6) [93] and performing 500 iterations with 500 replicates for bootstrap analysis [94]. We selected only linear and quadratic features classes because in general we had species with few records [95]. With the ENM evaluate function we obtained the Area Under the Curve (AUC) of the Receiver Operating Characteristic (ROC) plot [96] based on the test data (AUC_TEST_). To quantify the degree to which models overfit the data, we calculated three metrics: (1) the difference between training and testing AUC (AUC_DIFF_), (2) the Minimum Training Presence 10 omission rate (OR_MTP_) and, (3) 10% training omission rate (OR_10_). The final best model was selected using the Akaike Information Criterion (AICc) [91]. Likewise, we used the platform NICHE TOOLBOX to obtain the values of partial-ROC [94,97] for each final model obtained. Unlike AUC, Partial-ROC allows a differential weighting of omission and commission errors and focuses on meaningful predictions for model evaluation. Finally, the obtained continuous models for the suitability conditions of species were converted into binary maps (presence/absence) considering a threshold value that maximized the True Skill Statistics (TSS). For that, we elected predictions corresponding to 5% of omission error.

### Spatial analyses

To evaluate how land use change affected the potential distribution of snake species in the Argentinian Dry Chaco, we performed an analysis considering two approaches: (a) total reduction area for the species and (b) estimating the reduction area per year from 1976 to 2017. We used a map of the expansion of the agricultural frontier from 1976 to 2017 for Argentinian Dry Chaco Forest (available at http://geoportal.idesa.gob.ar/). For this step, we considered only the natural intact forest areas, whereas disturbed areas (i.e. urban areas, deforested areas, farming areas, and pastureland for cattle ranching) were not included [98,99]. Then, we assessed the importance of PAs for the obtained models calculating (in km^2^) the proportion of potential distribution areas within the current PAs system. Shape file of PAs were downloaded from the National Geographic Institute (IGN-ARGENTINA, available in http://www.ign.gob.ar/). Likewise, to assess if the current PAs system contained the most suitable bioclimatic conditions for the species, we performed a Kolmogorov-Smirnov (KS) test in R [100] verifying if values differed significantly between within and without protected areas [101].

### Spatial conservation prioritization analyses

We identified conservation priority areas using ZONATION 4.0 Software [40]. ZONATION algorithms generate a hierarchical priority ranking of the landscape based on the biological values of the spatial units (cells). Overall, ZONATION uses a raster for each biodiversity feature (herein snake species and anthropic variables), where each pixel contains information of either the occurrence or intensity of each feature [102]. The way the value of ‘loss of conservation’ is aggregated across features (occurring in a pixel) depends on the so-called ‘cell-removal’ rules. In this sense, the software produces a complementarity-based and balanced ranking of conservation priority over the entire landscape maximizing the species' occurrence and considering the different ‘penalization’ variables used [40,102]. For a more detailed explanation about the use of ZONATION see Di Minin et al. [102]. In this work, we compared scenarios with two different options of marginal aggregate loss: (1) the Additive Benefit Function (ABF), which emphasizes species richness minimizing extinction risk; and (2) the Core Area Zonation (CAZ), which emphasizes areas with both the highest suitability scores and the lowest uncertainty values for each species [102–104].

During the priority analysis, Zonation considers species priorities (weights), land cost, habitat quality, measures of connectivity, etc. [69,105,106]. We used as biodiversity features the probability of occurrence maps for each snake species and we weighted species assigning a high value (5) for threatened species and a low value (1) for those categorized as low conservation concern according to the IUCN (2018) and Giraudo et al. [53]. To promote the selection of optimal areas for current PAs expansion, we used as a hierarchical mask [102] a layer of national and provincial PAs (IUCN and UNEP-WCMC 2012). In this sense, the program identifies the best part of the landscape for an optimal and balanced expansion of existing PAs (which are preferably selected as the first option in the analysis), and also to compensate for specific ecological losses and satisfy the targets with minimum cost [102]. Given that most snake species cannot adequately be protected inside highly modified areas, we assigned negative weights or ‘penalization’ value to pixels covered by crops or urbanized areas. This last step prevented the software from selecting highly modified areas and assigning high conservation values to such areas. In this sense, considering that human influence tends to diminish habitat quality, and therefore, the potentiality for conservation, we used the Argentina Human Footprint layer [107] as a negative variable, ‘penalizing’ those pixels with high human influence. This Human Footprint layer (HII) was created considering data layers of human population pressure, human land use and infrastructure, and human access [108]. We assigned negative weights to these features (i.e. pixels in highly modified areas) so that the sum of the positive and negative weights was zero, allowing a balanced solution for prioritization [104,109]. In order to evaluate the influence of expansion of the agricultural frontier in the largest area of the Dry Forest, determining the relative importance of current PAs and identify complementary priority conservation areas for long-term protection of endemic snakes, we performed four alternative analyses in ZONATION. For the first priority areas analysis, we used only all snake species distributions, equally weighted, to obtain information about the most important areas to conserve in an ideal scenario without deforestation and fragmentation habitat. Then, in a second approach, we did an analysis considering all species distributions, also equally weighted, but with the PAs as hierarchical mask, which allowed us to identify where complementary areas to PAs occur, assuming fragmentation and habitat loss were not yet a current problem for the species. Thirdly, to identify the best opportunities for an optimal and balanced expansion of existing PAs which compensates specific ecological losses and satisfies the targets with minimum cost, we developed a priority areas analysis considering all species distributions, individual weighted according to the IUCN status, with the PAs as hierarchical mask and using the Human Footprint layer as a negative variable. Finally, to determine the relative importance of current PAs within the species distribution considering the expansion of the agricultural frontiers, we performed an analysis based on species distribution, individual weighted, using the Human Footprint as negative variable but without the PAs as hierarchical mask. After running these prioritization analyses, we plotted performance curves for all approaches considering the general patterns. These performance curves quantify the proportion of the original occurrences retained for each biodiversity feature at each top fraction of the landscape selected for conservation [40,102]. Finally, we determined the representativeness of (1) the current PAs network and (2) the top priority 17% of the landscape under protection (this percentage represents the AICHI targets; [110]).

## Results

We obtained 706 historical occurrence records for the 12 focal endemic snake species from which 360 independently records were used in ENMs (Table 1; S1 Table). The number of occurrence records used for each species and the percentage of contribution for bioclimatic variables in the models are summarized in Table 1. Overall, we observed that the maximum temperature of the warmest month (BIO 05) was the most important variable for almost all species (except *E*. *albertguentheri* and *P*. *baroni*). In these latter two species, precipitation seasonality (BIO 15) and diurnal temperate range (BIO 02) emerged as the most relevant variables respectively.

**Fig 1:**
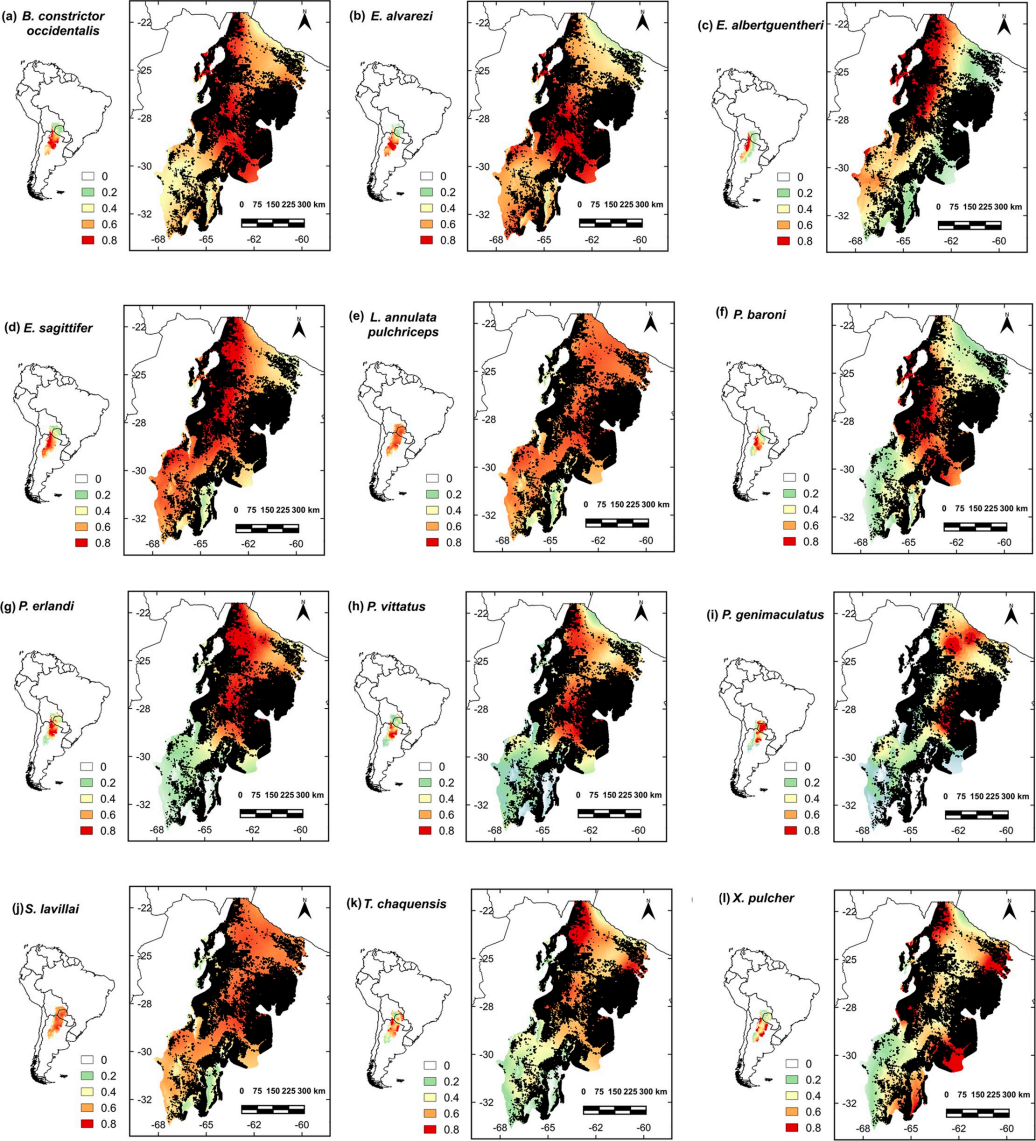
ENMs for snake species in the Argentinian Dry Chaco considering deforestation since 1976 to 2017. Warmer colors (red, orange and yellow) indicate higher habitat suitability while colors from green to white indicate the lowest predicted values. Black color indicates deforested areas. (a) *B*. *constrictor occidentalis*; (b) *E*. *alvarezi*; (c) *E*. *albertguentheri*; (d) *E*. *sagittifer modestus*; (e) *L*. *annulata pulchriceps*; (f) *P*. *baroni*; (g) *P*. *erlandi*; (h) *P*. *vittatus*; (i) *P*. *genimaculatus*; (j) *S*. *lavillai*; (k) *T*. *chaquensis*; (l) *X*. *pulcher*.

In general, models for each snake species performed well against the validation data (Fig 1 and Table 2). The Partial ROC bootstrap tests indicated significant ratio values (mean AUC ratios ≥1.4), low standard deviations and significant p-values (p <0.001) in all the models. Likewise, the Jackknife test (considering the ROC plot [96] based on the test data [AUC_TEST_]) showed that models also tended to be statistically significant (p < 0.01) for those species with <10 occurrence records. These results demonstrate that all models performed better than random (Table 2), and, thus, performed well in estimating potential distributions of species.

**Table 2:** Mean AUC_TRAIN_, AUC_TEST_, AUC_DIFF_ and Partial ROC (AUC_RATIO_) for snake species models with the minimum AICc value.

Species	FC	RM	AUC_TRAIN_	AUC_TEST_	VarianceAUC_TEST_	AUC_DIFF_	VarianceAUC_DIFF_	OR_MTP_	OR_10_	Mean value for AUC_RATIO_	sd AUC_RATIO_
***B*. *constrictor occidentalis***	LQ	2.2	0.8721	0.8539	0.0027	0.0234	0.0023	0.0472	0.1806	1.5722	0.05
***E*. *alvarezi***	LQ	4.6	0.9136	0.8933	0.0015	0.0226	0.0017	0.0571	0.1762	1.8145	0.05
***E*. *albertguentheri***	L	2.2	0.9357	0.9334	0.0016	0.0122	0.0008	0.0667	0.1333	1.9626	0.02
***E*. *sagittifer modestus***	L	2.2	0.8657	0.8494	0.0037	0.0250	0.0031	0.0444	0.1556	1.4922	0.02
***L*. *annulata pulchriceps***	LQ	4.6	0.7969	0.7969	0.0065	0.0184	0.0041	0.0667	0.1333	1.6301	0.06
***P*. *baroni***	LQ	1	0.9276	0.9182	0.0005	0.0101	0.0007	0.0154	0.1462	1.5683	0.11
***P*. *erlandi***	L	1	0.8738	0.8555	0.0060	0.0324	0.0045	0.1133	0.1867	1.5954	0.07
***P*. *vittatus***	L	1	0.9169	0.8982	0.0069	0.0343	0.0033	0.1000	0.1400	1.7256	0.13
***P*. *genimaculatus***	L	1	0.9182	0.8976	0.0283	0.0545	0.0161	0.3333	0.5000	-	-
***S*. *lavillai***	LQ	2.2	0.7958	0.7534	0.0692	0.0848	0.0478	0.2500	0.3750	-	-
***T*. *chaquensis***	LQ	4.6	0.7946	0.8040	0.0246	0.0365	0.0044	0.2857	0.2857	-	-
***X*. *pulcher***	LQ	1	0.8733	0.8593	0.0155	0.0421	0.0075	0.0143	0.1229	1.4737	0.02

FC: feature class (L = linear, Q = quadratic); RM: regularization multiplier; AUC_TRAIN_ and AUC_TEST_: Area Under the Curve based on train and test data; Variance AUC_TEST_; Variance AUC_DIFF_: the difference between training and testing AUC; OR_MTP:_ Minimum Training Presence omission rate (OR_MTP_); OR_10_: 10% training omission rate; AUC_RATIO:_ partial-ROC.

**Fig 2:**
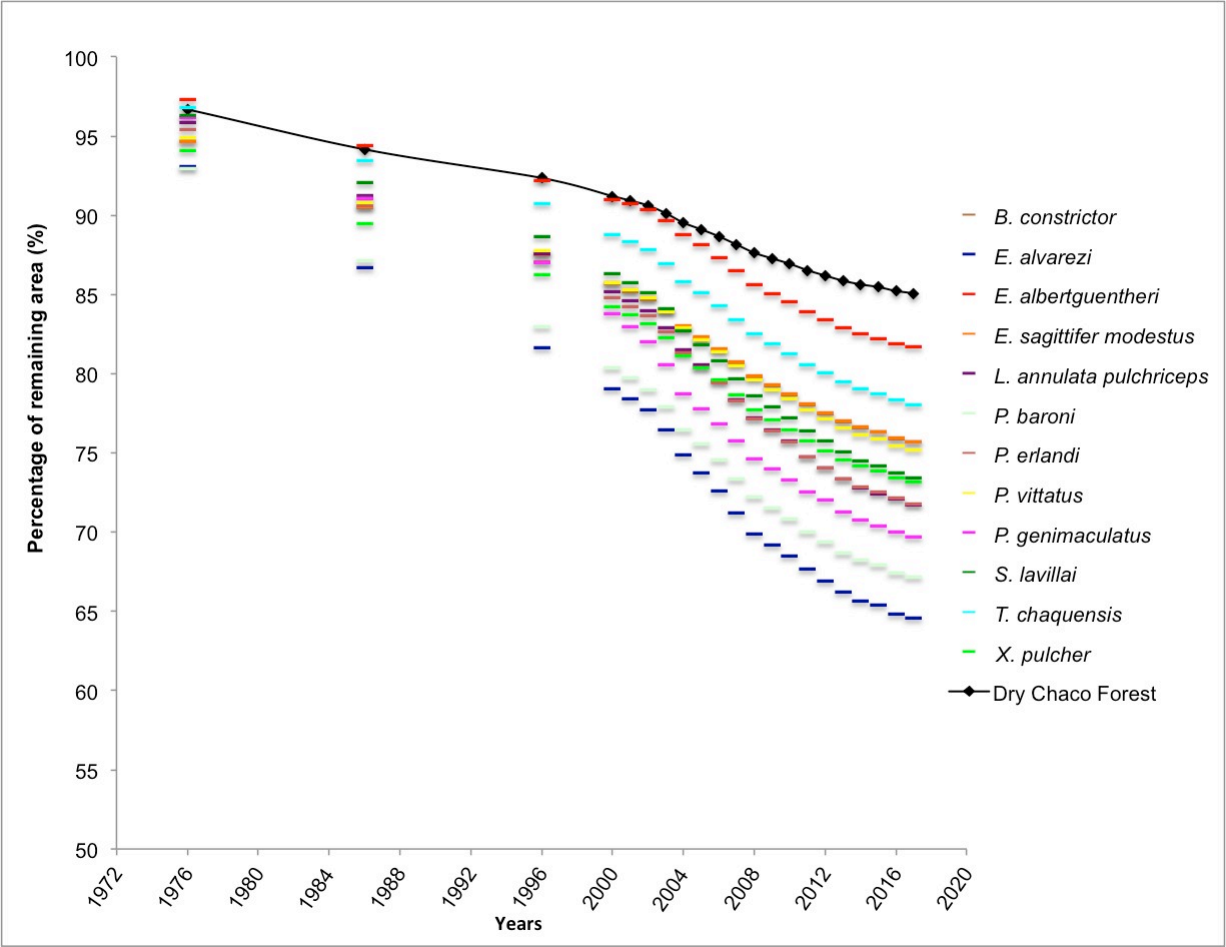
Percentage of the remaining distributional area for snake species and for the Argentinian Dry Chaco forest from 1976 to 2017.

Argentinian Dry Chaco has experienced a 15% reduction in area between 1976 (786,790 km^2^) and 2017(669,410 km^2^) (Fig 2). We observed that models predicted, on average, a distributional range extent of ~447,000 km^2^ for focal species (min 204,828 km^2^ [*E*. *alvarezi*]–max 748,630 km^2^ [*P*. *vittatus*]), with at least five species (*B*. *constrictor occidentalis*, *E*. *sagittifer modestus*, *P*. *vittatus* and *X*. *pulcher*) with large areas of distribution (>500,000 km^2^). The two most spatially restricted species were *E*. *alvarezi* and *L*. *annulata pulchriceps* (Table 3). Likewise, we observed that Argentinian Dry Chaco represent, on average, ~74% of species distribution species with only *P*. *genimaculatus* and *T*. *chaquensis* with less than 60% of distribution within Argentinian Chaco. However, when we considered the effects of fragmentation and habitat loss, we observed an average ~73% reduction (min 64.56%[*E*. *alvarezi*]–max 81.72% [*E*. *albertguentheri*]) in suitable areas for snakes species in the Dry Chaco forest. The three species with most habitat loss estimated were *E*. *albertguentheri*, *T*. *chaquensis*, and *E*. *sagittifer modestus*; while that snakes less affected were *P*. *genimaculatus*, *P*. *baroni*, *E*. *alvarezi*. The general trends, from 1975 to 2019, for the reduction in areal extent for the Argentinian Dry Chaco forest and species' potential distribution are summarized in the Fig 2.

**Table 3:** Potential distribution area (km^2^) of snake species in the Dry Chaco forest.

Species	Mean (max.tss)	Chaco area (km^2^)	Argentinian Chaco area (km^2^)	% within PAs (Argentina)
***B*. *constrictor occidentalis***	0.4325	531,585	480,379	3.67
***E*. *alvarezi***	0.5647	204,828	200,603	0.21
***E*. *albertguentheri***	0.4658	388,267	316,548	2.14
***E*. *sagittifer modestus***	0.3886	695,184	480,195	3.07
***L*. *annulata pulchriceps***	0.6394	274,455	239,425	2.83
***P*. *baroni***	0.3772	342,248	325,599	2.74
***P*. *erlandi***	0.4074	440,746	311,817	3.87
***P*. *vittatus***	0.4217	748,630	452,364	3.92
***P*. *genimaculatus***	0.5300	321,559	109,622	5.90
***S*. *lavillai***	0.6491	363,658	236,147	2.21
***T*. *chaquensis***	0.6459	489,898	291,929	4.62
***X*. *pulcher***	0.3265	570,273	430,350	4.00

Also shown is the threshold value given by the True Skill Statistics (Mean max.tss) and the percentage of the distribution within Argentinian protected areas (PAs).

**Fig 3:**
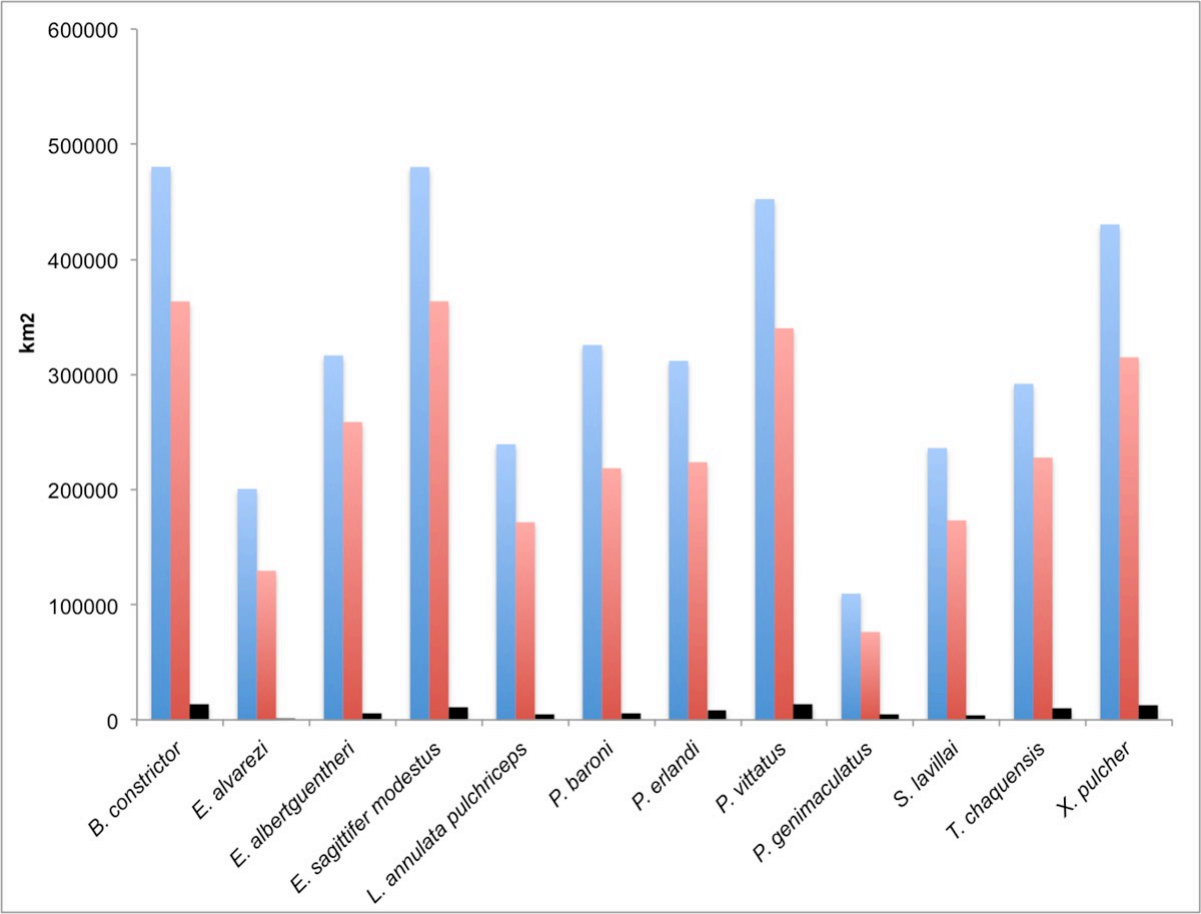
Potential distribution areas (in km^2^) of snake species in Dry Chaco forest. Blue bars: distributional range predicted for each snake species in the Dry Chaco forest; Red bars: distributional range predicted for species in Argentinian Dry Chaco forest considering habitat loss/deforestation; Black bars: distributional range predicted within protected areas.

Using IUCN criteria, all of our focal snake species are not considered to be threatened or have not been evaluated (Table 4) [53]. Yet, we found that only a small portion of snakes’ ranges occur within existing PAs from Argentina (Table 4, Fig 3). On average, we observed that 3.27% (min: 0.21 [*E*. *alvarezi*]–max: 5.9 [*P*. *genimaculatus*] %) of predicted distributions by species are within some category of protection. Also, for all focal species, the areas inside PAs had significantly lower suitability values compared with the areas outside PAs (Table 4).

**Table 4:** Comparison between the suitability values (Mean± SD) outside and inside PAs.

Species	Outside PAs Mean± SD	Inside PAs Mean± SD	Kolmogorov-Smirnov test	Giraudo et al. (2012)	IUCN (October 2018)
***B*. *constrictor occidentalis***	0.6361±0.1343	0.5761±0.1589	D = 0.2689, p < 0.000	AM	Not evaluated
***E*. *alvarezi***	0.6368±0.1289	0.5585±0.1463	D = 0.2765, p < 0.000	AM	Not evaluated
***E*. *albertguentheri***	0.4579±0.2596	0.4368±0.3001	D = 0.1915, p < 0.000	NA	LC
***E*. *sagittifer modestus***	0.6385±0.1405	0.5285±0.1842	D = 0.3741, p < 0.000	NA	LC
***L*. *annulata pulchriceps***	0.6205±0.1018	0.5093±0.2418	D = 0.2988, p < 0.000	NA	Not evaluated
***P*. *baroni***	0.4956±0.2275	0.4561±0.2418	D = 0.0988, p < 0.000	IC	Not evaluated
***P*. *erlandi***	0.5101±0.2669	0.3781±0.2921	D = 0.2303, p < 0.000	NA	LC
***P*. *vittatus***	0.4978±0.2582	0.3293±0.2533	D = 0.2621, p < 0.000	-	Not evaluated
***P*. *genimaculatus***	0.1508±0.1199	0.0886±0.0987	D = 0.2686, p < 0.000	IC	Not evaluated
***S*. *lavillai***	0.6067±0.1141	0.4879±0.1795	D = 0.3017, p < 0.000	NA	Not evaluated
***T*. *chaquensis***	0.5112±0.2091	0.3792±0.2609	D = 0.2784, p < 0.000	NA	Not evaluated
***X*. *pulcher***	0.5532±0.2016	0.5615±0.2164	D = 0.1197, p < 0.000	NA	LC

Also shown are the values of Kolmogorov-Smirnov (KS) test and the Argentine categorization [53] and conservation status (the IUCN Red List of Threatened Species- October 2018) of snakes in the Dry Chaco forest. AM: Threatened; IC: Insufficiently Known; NA: Not Threatened; LC: Least Concern.

**Fig 4:**
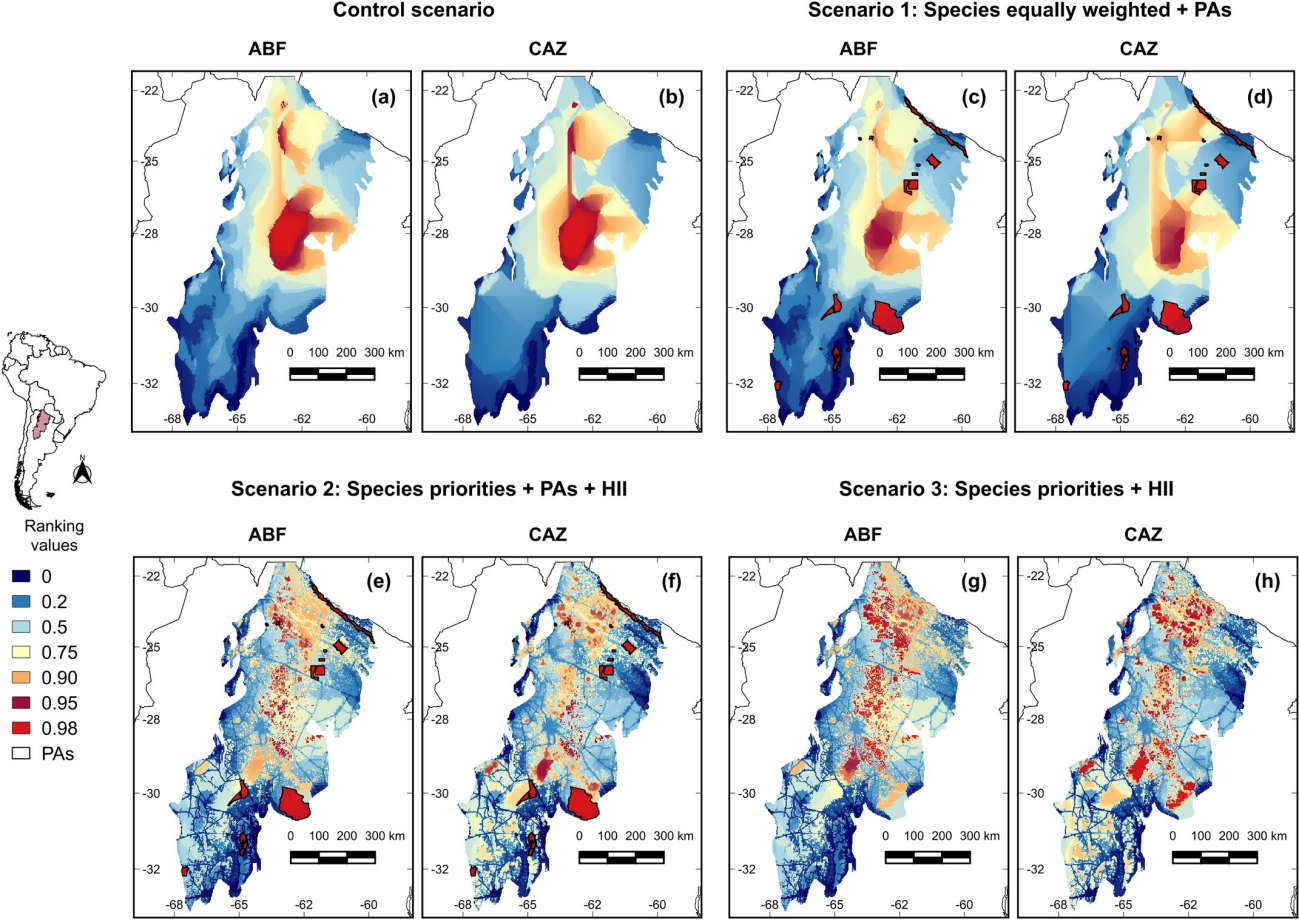
Priority areas for focal snakes distributed in the Argentinian Dry Chaco under different prioritization scenarios. In all scenarios we compared the outputs that emphasized species richness minimizing extinction risk [Added Benefit Function, ABF (left)] and emphasized areas with both the highest suitability scores and the lowest uncertainty values for each species [Core Area Zonation CAZ (right)]. (a-b) Control scenario (species equally weighted); (c-d) Scenario 1: species equally weighted + PAs; (e–f) Scenario 2: species individual weighted + PAs + cost (Human Footprint—HII); (g-h) Scenario 3: species individual weighted + cost (HII). The color scheme shows the nested ranking on a map. Ranking the biological value of the site: 2% high priority (light red); 2–5% (dark red); 5–10% (orange); 10–25% (yellow); 25–50% (light blue); and 50–80% (blue); 80–100% (or the least valuable 20%) (dark blue).

**Fig 5:**
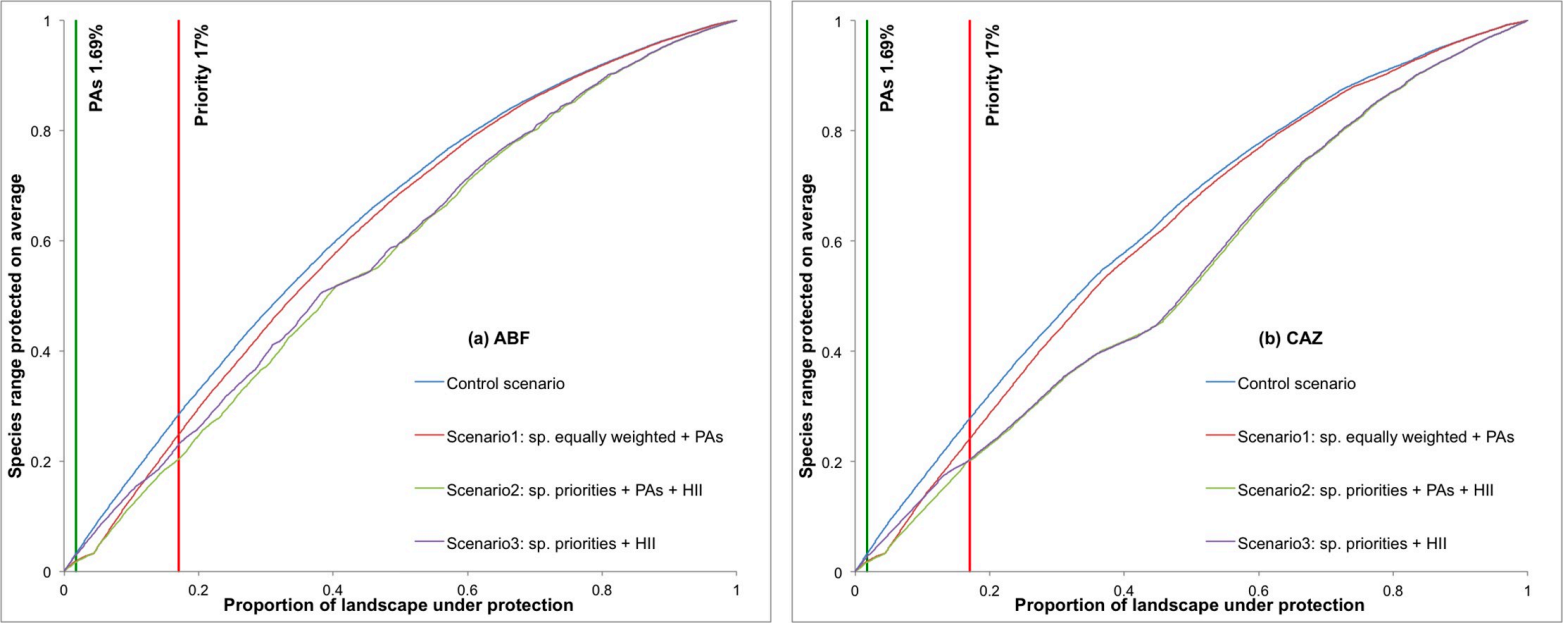
**Performance curves of the spatial prioritization solutions for both removal rules ABF [Added Benefit Function] (a) and CAZ [Core Area Zonation] (b).** Each line represents a scenario [Control scenario: species equally weighted; Scenario 1: species equally weighted + PAs; Scenario 2: species individual weighted + PAs + cost (Human Footprint-HII) and; Scenario 3: species individual weighted + cost (HII)] showing the proportion of landscape protected and its corresponding average species range protected. The vertical lines represent current proportion of land in PAs (1.69%) and the recommended AICHI target (17%) [110].

**Fig 6:**
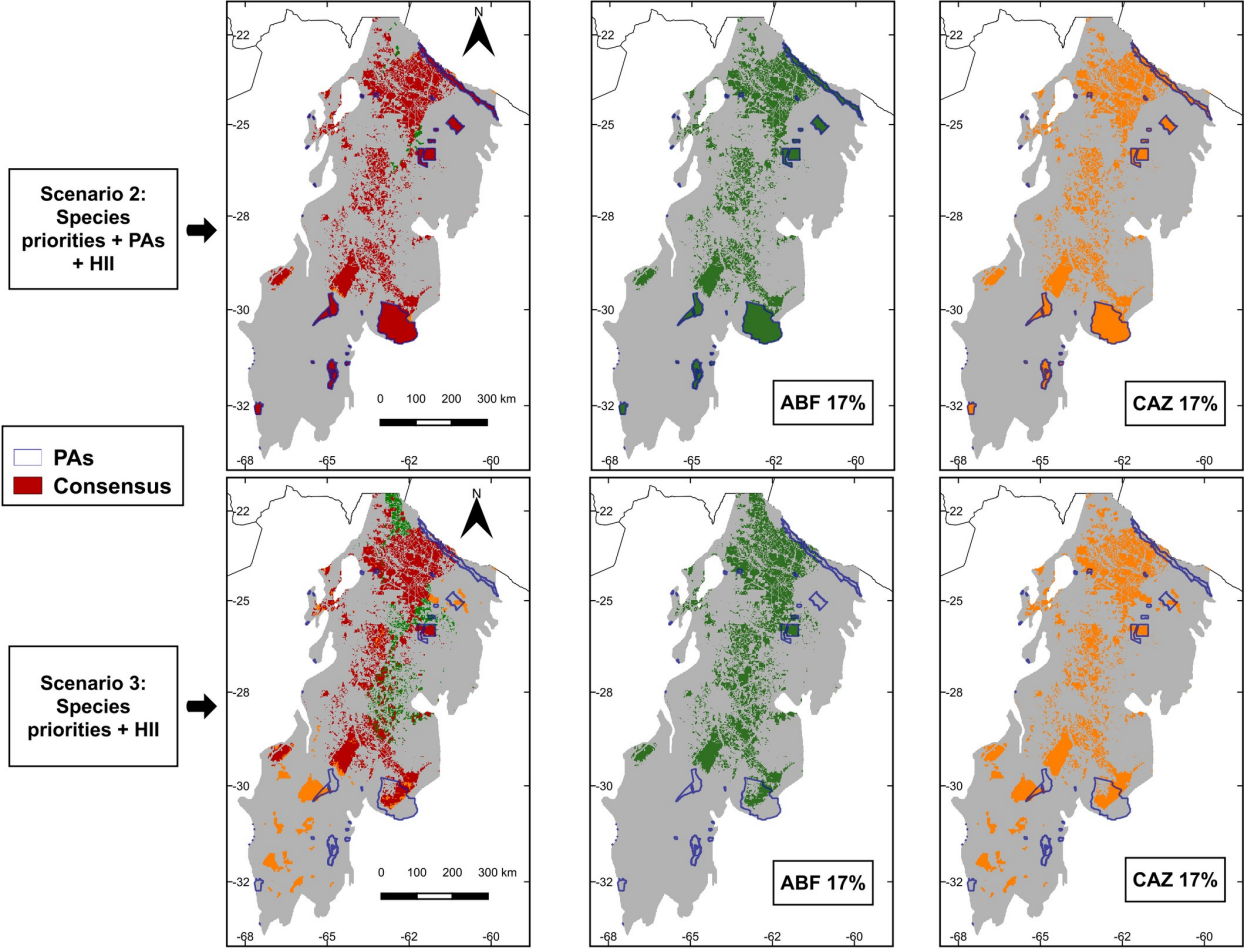
Maps showing the 17% of priority conservation for ABF (Added Benefit Function) and CAZ (Core Area Zonation) in Scenario 2 [Species individual weighted + PAs + cost (Human Footprint-HII)] and; Scenario 3 [Species individual weighted + cost (HII)]. Red pixels correspond to the consensus areas selected by both CAZ and ABF prioritization schemes for conservation snakes and which also complement current PAs system of the Argentinian Dry Chaco forest. Those selected by only one prioritization scheme are shown in green (ABF) or orange (CAZ).

Prioritization scenarios for snake species based on ZONATION analyses are shown in Fig 4. As a general pattern, the priority areas change when the cost of the HII is considered. The current PAs cover 1.69% of the Argentinian Chaco forest. In all scenarios, this percentage represented on average ~3% (max. Control scenario: 2.99% in both ABF and CAZ; min. Scenario 2: 1.58%ABF and 1.56%CAZ) of the predicted distributional ranges of snakes. When we modeled the AICHI landscape goals of 17% of area protected, all snake species benefited markedly from increase in protected areas. The value, not surprisingly, was highest (~28%) in the scenario without deforestation and lower under more realistic scenarios that incorporated land use change and species priorities [Scenario 2: 20.38% ABF and 20.19% CAZ] (Fig 5A and 5B). Further, the representativeness of distribution of snakes’ species was greatest in HII scenarios [Scenario 3: 23.05% ABF and 20.32% CAZ] (Fig 5A and 5B). Moreover, considering a 17% protection goal, the priority areas identified in Scenario 2 showed spatial patterns consistent for both algorithms and slightly different to that from Scenario 3, which included more priority areas in the center (ABF) and south (CAZ) of the Dry Chaco forest (Fig 6). Finally, consensus areas among scenarios can be found adjacent to current PAs while other PAs are not found to be adjacent with priority selected areas regardless of model scenario (Fig 6).

## Discussion

Here we present for the first time how expansion of the agricultural frontier has potentially impacted the distribution of endemic snake species in the Dry Chaco forest. Further, we evaluate the degree with which those species are represented in the current protected area system in the Argentinian Chaco and propose locations of high priority areas for snake conservation in the region. In the paragraphs below we discuss some of the key results including the reduction of potentially suitable habitat for snakes, but also the potentially limited value of the current protected area system to conserve quality habitats for endemic snakes of Dry Chaco forest.

### Impacts of deforestation and expansion of the agricultural frontiers

The Dry Chaco is severely threatened due to the significant transformation of forest for agriculture and timber production. In the last decades, the growing demand for agricultural products and the new “extreme weather resistant” variety of crops have led to an exponential increase in areas under crop production [22,111]. In Argentina, which harbors a large portion of Dry Chaco forest, deforestation alone has resulted in a 15% reduction (117,380 km^2^) in the past four decades (Fig 2). Less easily measured is the percent of Dry Chaco areas, which have not been transformed, but may have experienced degradation due to escaped fires, logging, introduction of invasive species, and other factors. These habitat loss and degradation processes, together with the limited percent of Chaco in protected status (1.69%), highlight the present need to direct conservation planning efforts that consider and evaluate the trade-offs between conservation and economic development.

Evaluating the impact of deforestation and expansion of agricultural frontier on snakes is hampered by the limited information on the natural history and distributions of many snake species in the Dry Chaco forest. Indeed, for the majority of our focal species, IUCN does not have sufficient information to categorize the conservation status of the species (Table 4). Particularly under such conditions, the use of ENMs to predict potential distributions has become a valuable tool in conservation planning, and has been used for other taxa in Argentinean Dry Chaco context [16,112]. Using results from ENMs, we demonstrated that the increase in deforestation rates in the Argentinian Dry Chaco over the last four decades have resulted in a disproportionate decrease in the distribution of nearly all snake species analyzed (Fig 1 and Fig 2). This decreasing distribution has also been demonstrated for *Leptodactylus* species (Anura) with at least 25% of their geographic range occurring in the Dry Chaco. In this work, the potential distribution area of these species decreased between 16% and 10% approaching with the deforestation rate from 1976 to 2013 [37]. Also, this loss was even more relevant for the distribution of species, which had already been transformed to agriculture in the last 40 years experienced. However, not all species respond in a similar way to forest loss and degradation as differences in natural history among the species may result in greater or lower risk. For example, generalist or fossorial snakes, such as *E*. *sagittifer modestus*, *P*. *vittatus* and *X*. *pulcher*, which tolerate a wide range of bioclimatic conditions, may be more able to adapt to transformed and degraded environments [46,113]. Nonetheless, these areas which likely include periurban or urban zones [47], can represent other risks as a result of negative interactions with humans which tend to fear and indiscriminately kill snakes [55,114]. In contrast, specialist snake species with restricted bioclimatic conditions, such as *B*. *constrictor occidentalis* and *E*. *alvarezi*, are expected to have increased vulnerability with changes in habitat conditions [46,113]. For example, studies have shown that loss of habitat affects body condition, clutch size and testicular volume of *B*. *constrictor occidentalis* [52,53]. Consequently, the natural history of the species is important to consider in management and conservation planning, but was beyond the scope of the current study.

### Role of argentinian protected areas for snake conservation

The current protected area system in Argentinian Dry Chaco is likely inadequate for long-term protection of most of the endemic snake species studied here for several reasons. First, only 1.69% of the Argentinian Dry Chaco forest is currently under protection. Despite this, the current PAs system represents, on average, 3.27% of snakes predicted distributions. Yet, this is still a very small amount of the range of the potential distribution in the Dry Chaco. In some cases, such as the threatened (AM) *E*. *alvarezi*, only 0.21% of their predicted range is includes in the PAs (Fig 3). Secondly, our results indicated that the areas inside the PAs generally contained lower suitability values compared with areas outside them (Table 4). Therefore, the current PAs not only provide very limited spatial protection for these endemic snakes, but also protect habitats of apparently less optimal bioclimatic conditions. These results are consistent with studies that have analyzed the role of PAs in the protection of other groups of vertebrates in the Chaco forest. For example, a study for 63 vertebrate species (21 amphibians, 20 mammals and 22 birds) with at least 70% of their distributions within the Gran Chaco, found that current PAs represent on average 9% of the total distribution of endemic species [47]; also, the PAs protects around 5% of the potential distribution of *Leptodactylus* species (Anura) [37]. Moreover, comparing the control map with the three alternatives scenarios (Fig 4), our spatial conservation prioritization revealed that the current PAs system of the Argentinian Dry Chaco did not capture the most suitable areas for endemic species of snakes, and in fact, were notable in their failure to align with more suitable locations.

The PAs in Argentina are apparently inadequate to protect these endemic snakes and other vertebrates, this is not surprising given that information on biodiversity and habitat were not used in the creation of the PAs. Further reducing the conservation value of these PAs is the increasing fragmentation of land adjacent to these lands, as well as poor legal-institutional security and weak or inexistent management programs [8]. Lack of planning and design in selection of PAs has resulted in inefficiency not only in the investment of human and financial resources but also in terms of conservation value of the PAs [8,30].

The prioritization analyses conducted in this study were consistent in identifying the northwestern and central parts of the Dry Chaco as high priority areas and the southern Chaco, where the forest is much more degraded due to anthropogenic actions [25,115], as the areas with the lowest priority ranking (Fig 4). These scenarios differed in a number of ways including whether or not they included information on current land use (i.e., incorporated a Human Footprint Index), included PAs in prioritization selection, considered species equally or ranked them according to threat status. Further, how areas were selected for each scenario was done in two ways–minimizing species extinction risk (ABF) and incorporating information about suitability of areas and uncertainty (CAZ). When we compared the performance curves of the different scenarios, the representativeness of the current PAs system covered, on average, only a small portion of the distribution of endemic snake species [Control: 2.99 (ABF) and 2.99 (CAZ); Scenario 1: 1.78% (ABF) and 1.72% (CAZ); Scenario 2: 1.58% (ABF) and 1.56% (CAZ); Scenario 3: 2.80% (ABF) and 2.50% (CAZ)] (Fig 5A and 5B).

We suggest that the most realistic scenario from which to draw conclusions and guide future conservation planning is Scenario 2, which incorporated species individual weighted + PAs + Human Footprint (HII) as a cost layer. This scenario results in the most realistic solution by selecting well-conserved Dry Chaco forest areas for snake conservation in Argentina (Fig 4E and 4F). When 17% AICHI targets for land protection were incorporated, this scenario conserved an average 20.38% (ABF) to 20.19% (CAZ) of snakes’ potential distribution; Scenario 3 presented similar result for CAZ outputs; while the other scenarios (Control and Scenario 1) accounted for above 20% of protection but ignoring current patterns of fragmentation and habitat loss (Fig 5A and 5B). For both ABF and CAZ outputs in Scenario 2, the highest areas of prioritization were located on continuous native forest remains, many of which are located in transitional areas between Dry Chaco and southern tropical Andean forest (i.e., Austral Yungas ecoregion). These transition zones with foothill forests may serve as corridors to connect patches of remaining native forest between the western Dry Chaco and Yungas forests. Currently, due to agricultural expansion, however, the connection between Yungas-Chaco has been reduced from 1,035 km to only 162 km [116–118] and, as a consequence, this transition zone is highly threatened [26,119]. Overall, our priority areas coincided with a spatial prioritization analysis made for the Gran Chaco using ZONATION [16]. However, in this study, reptiles were not considered, thus our results complement and highlight the relevance of these areas.

The adequacy of the current PAs for snakes conservation is highlighted by comparing priority area selection between Scenarios 2 and 3 (Fig 6). While selection is constrained to complementing PAs in Scenario 2 (i.e., adds onto existing PAs), no such constraint exists in Scenario 3. As a consequence, there is less overlap of priority areas with current PAs in Scenario 3. This likely occurs because of generally lower suitability values for snakes found inside protected areas (Table 4). Further, releasing the constraint of parks in selecting priority areas appears to increase the sensitivity to selection rules (ABF and CAZ) although considerable consensus occurs between the two (Fig 6). This difference is likely also a result of the importance of considering suitability in site selection and the existing human impact in the current PAs system. According to that, Argentinian government should invest economic resources to working in the strengthening of action plans and management of the PAs. Therefore, these results highlight the need to not only conserve existing protected areas but also expand the current PAs to complement and update the current environmental policies.

Our results, while highlighting several areas of concern and making some specific recommendations, have a number of limitations. Like other studies that have applied species distribution models, our study also is limited by (1) lack of data for some species, (2) potential geographic bias of data and, (3) lack of true absences [86,90,96]. Although we have worked on solving these issues, we recognize the limitations presented by the low number of records for some species. In our approach, we evaluated each model with parameters (feature classes and regularization multiplied) designed to reduce model complexity and improve model performance.

### Final considerations

The Chaco forest is one of the most threatened ecosystems in the world, with a high rate of deforestation and fragmentation [16]. The spatial pattern of human activity is a relevant factor in conservation planning. With increasing human activity, the possibilities to adequately conserve biodiversity are decreasing [120]. However, our spatial prioritization approach demonstrates that it is still possible to complement the current protected areas network and promote habitat connectivity. Our finding identifies spatial priorities that minimize conflicts with human activities, a key issue in a threatened ecoregion characterized by the rapid transformation of natural areas due to the advancing agricultural frontier. Although this study was carried out taking into account the agricultural expansion in Argentina, it should be noted that our prioritization results identified high priority areas located near Bolivia and Paraguay. The current situation of the Chaco forest in Bolivia and especially in Paraguay is quite similar to Argentina [16,121]. Further study should extend this analysis into Bolivia and Paraguay and include more taxa, climate change scenarios, regional habitat loss and remaining habitat connections in the entire Gran Chaco region [16,20,29,36,37,52]. In addition, collaborative work among countries is urgently needed to complement existing protected areas and to generate comprehensive plans for expanding and connecting priority areas in the whole ecosystem. We need immediate concrete actions taken by stakeholders and decision-makers, or, we will risk losing the best opportunities to conserve the biodiversity of the Dry Chaco forest.

## Supporting information

S1 Table Records of snakes species for Dry Chaco forest after filtering. (XLS)
